# Comprehensive analysis of hypoxia-related genes for prognosis, immune features, and drugs treatment strategy in gastric cancer using bulk and single-cell RNA-sequencing

**DOI:** 10.1038/s41598-022-26395-5

**Published:** 2022-12-16

**Authors:** Guoqiang Tao, Chengwen Jiao, Yong Wang, Qi Zhou

**Affiliations:** Department of General Surgery, Shanghai Punan Hospital, NO. 279 Linyi Road, Pudong New District, Shanghai, China

**Keywords:** Biomarkers, Molecular medicine, Oncology

## Abstract

Hypoxia is one of the malignant characteristics of solid tumors and is related to the multiple malignant characteristics of the tumor. No study has not yet reported a systematical analysis of the characteristics of hypoxia from single-cell resolution in gastric cancer. In our research, we investigated the hypoxia features of various types of cells in single-cell resolution, identified hypoxia-related genes by the weighted gene co-expression network analysis method. Through the hypoxia-related genes from single-cell levels, we screened out 13 genes and established a prognostic model. This model performs well in the training dataset and multiple independent verification data sets. We thought that tumor hypoxia might affect the DNA methylation of cells and promote the transcription of genes associated with malignant features, thereby promoting tumor progression. We found that the more tumor associated genes in the high-risk group showed hypomethylation and high hypoxia-risk score group have more tumor-related genes, more immunosuppressive immune cells and more enrichment of cancer -related pathways. The lower risk group is more sensitive to three chemotherapy drugs for gastric cancer. Our study illustrates the crucial role of hypoxia in gastric cancer. Hypoxia-related gene prognostic model has been established and has good performance. Hypoxia-related risk score can also be used to guide a patient’s drug treatment strategy.

## Introduction

Gastric cancer (GC) is the sixth most common cancer and the third leading cause of cancer-related deaths worldwide^1^. Surgical resection and adjuvant chemotherapy have been considered the mainstay of treatment for GC in recent decades. However, many GC patients are always diagnosed at an advanced stage, which severely limits the therapeutic effect. Thus, novel prognostic classifiers or therapeutic biomarkers are urgently needed to improve the clinical benefits of GC patients.

Hypoxia is one such unfavorable environment that can impair tumor function. Conversely, hypoxia prompts tumors to develop more characteristic malignant behavior^2^. Hypoxia can promote the formation of new blood vessels by inducing Hypoxia-inducible factor 1-alpha (HIF-1a)^3^, Vascular endothelial growth factor (VEGF)^4^, C–C Motif Chemokine Ligand 28 (CCL28)^5^ and other cytokines. Hypoxia also affects the immune system through multiple pathways, such as induction of transcription factors or target genes to suppress T cell proliferation, and induction of mitochondrial stress to promote T cell exhaustion^6,7^. Therefore, we speculate that hypoxia-related features can be used to predict prognosis and drug treatment response.

Because tumor tissue is composed of a variety of cells, we used single-cell sequencing data to identify gene signatures associated with hypoxia in epithelial cells. The differences between high and low hypoxia scores were investigated from the aspects of genome, DNA methylation, and tumor-infiltrating immune cells. We then explored the possibility of hypoxia score predicting response to chemotherapy drugs.

## Result

### Characterization of hypoxia in single cell resolution

**Figure 1:**
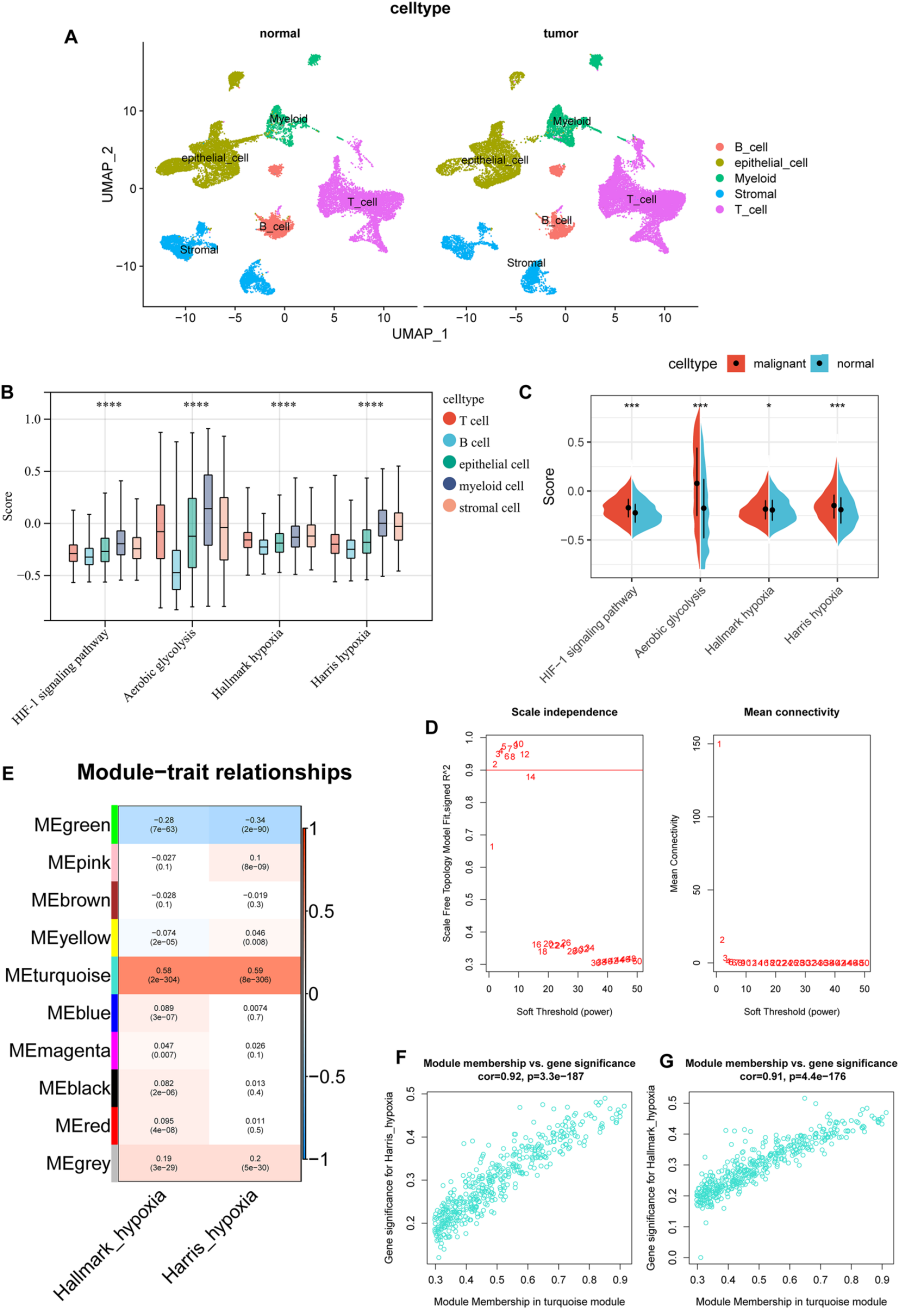
(**A**) The UMAP plot of cells in normal tissue and tumor tissue, which are color-coded based on their associated clusters. (**B**) Differences among the four hypoxia-related pathways among the five types of cells. (**C**) Differences among the four hypoxia-related pathways among malignant and non-malignant epithelial cells. (**D**) Analysis of the scale‐free fit index and the mean connectivity for various soft‐thresholding powers. (**E**) Table cells showing Pearson’s correlation coefficients and corresponding P-value between module eigengenes (ME) and the variables. (**F,G**) Scatter plots of the gene significance and module membership in turquoise module. The x-axis indicates the module membership (MM) which quantify how close a gene is to a given module. The y-axis indicates the gene significance (GS) which is correlated with clinical trait.

Since the GSE183904 single-cell dataset contains a large amount of single-cell sequencing data, we only selected all normal gastric tissue sequencing samples and part of gastric cancer sequencing samples (Details of picking samples in Table S1). After quality control processing, 43,956 cells remained for further analysis. Annotating all cell clusters according to marker genes, we roughly divide all cells into 5 categories (epithelial cells, T cells, B cells, stromal cells and myeloid cells) (detailed markers are in Table S2, Fig. 1A and Fig. S1). After extracting the expression matrix of all cells in the tumor tissue and calculating the enrichment scores of hypoxia-related gene sets, we found that there were significant differences in the enrichment scores of the four hypoxia-related gene sets of the five types of cells (Fig. 1B). Therefore, separate analysis of different types of cells is necessary. We further investigated and found that 5 cell types differed in 50 hallmark gene sets (Fig. S2). We found that most hallmark gene sets associated with tumors were enriched in epithelial, myeloid, and stromal cells. Among them, DNA repair, glycolysis, Notch signaling, and p53 pathway have higher levels in epithelial cells (Fig. S2). We then analyzed epithelial cells alone and found that epithelial cells in tumor tissues were significantly enriched for hypoxia-related signature genes compared to normal tissues (Fig. 1C).

### WGCNA

Due to the limitation of single-cell sequencing technology, the single-cell expression is sparse, so we only used the first 5000 hyper-variable genes for weighted gene co-expression network analysis (WGCNA). A soft threshold = 2 was selected to construct a scale-free network (Fig. 1D). A total of 9 gene modules were identified after setting the minimum cluster size as 50 (Fig. 1E). The turquoise module exhibited the highest correlation with hallmark hypoxia (R = 0.58, P = 2e − 304) and Harris hypoxia (R = 0.59, P = 8e − 306) (Fig. 1E). Scatter plots of module membership and gene significance relationships also demonstrated a high correlation between turquoise module and two hypoxia scores (Fig. 1F,G). Finally, we obtained 331 hypoxia-related genes and found they are significantly enriched in cancer-related pathways, such as: Focal adhesion, ECM-receptor interaction, PI3K-Akt signaling pathway, Pathways in cancer and so on (Tables S3, S4).

### Establishment and validation of hypoxia-related prognostic signature for overall survival in gastric cancer

**Figure 2:**
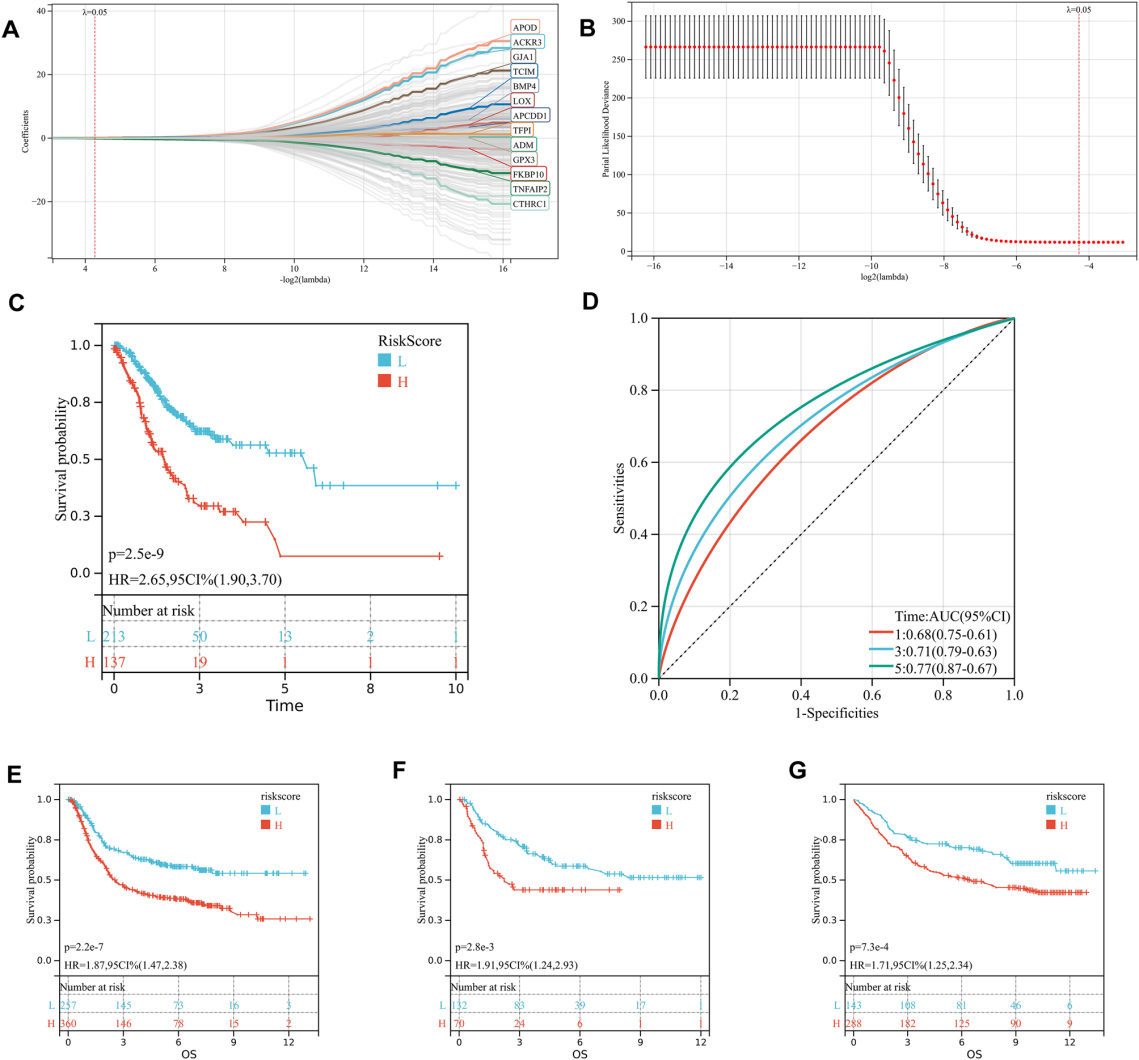
Construction of hypoxia-related prognostic model. (**A,B**) Partial likelihood deviance for the lasso regression and Lasso regression analysis. (**C,D**) Patients were divided into high-risk and low-risk subgroup based best cutoff, Kaplan–Meier analysis demonstrated that patients with higher hypoxia-related risk score exhibited worse overall survival in TCGA-STAD, ROC curves showing the predictive efficiency of the model on the 1-, 3-, and 5-years survival rate. (**E–G**) The prognostic difference was validated in 3 independent cohorts.

First, we performed log2 processing on the expression matrix of TCGA-STAD, and then performed z-score transformation. Subsequently, LASSO Cox algorithm was applied to identify the most robust prognostic genes. The optimal λ value of 0.0516241010727967 was selected (Fig. 2A,B). Finally, hypoxia-related risk score formula was established as follows: hypoxia-related risk score (HRRS) = 0.00178212549926764*ACKR3 + 0.046675316980481*ADM − 0.0699862245428993*APCDD1 + 0.0823475442114259*APOD-0.018912760227784*BMP4 + 0.0347669705181442*CTHRC1 + 0.0324360308876823*FKBP10 + 0.0723308062908515*GJA1 + 0.0525503764134501*GPX3 + 0.0118404129802321*LOX + 0.0378815306209615*TCIM + 0.0627550544666857*TFPI-0.0889186008713304*TNFAIP2. The Kaplan–Meier (KM) plot demonstrated that the high HRRS group had unfavorable overall survival (OS) compared with the low-HRRS group (P = 2.5e − 9, Fig. 2C). Moreover, the area under the curve (AUC) for 1-year, 3-year and 5-year OS were 0.68, 0.71 and 0.77 (Fig. 2D), respectively, which were good classification results. Then, the prognostic value of HRRS was validated in three independent cohorts (GPL570 metadata set: HR = 1.87, 95% CI  1.47–2.38, P = 2.2e − 7; GSE26942: HR = 1.91, 95% CI  1.24–2.93, P = 2.8e − 3; GSE84437: HR = 1.71, 95% CI  1.25–2.34, P = 7.3e − 4; Fig. 2E–G). 13 genes are related to prognosis (Fig. S3).

### Construction of integrated models to optimize risk stratification and survival prediction in gastric cancer

**Figure 3:**
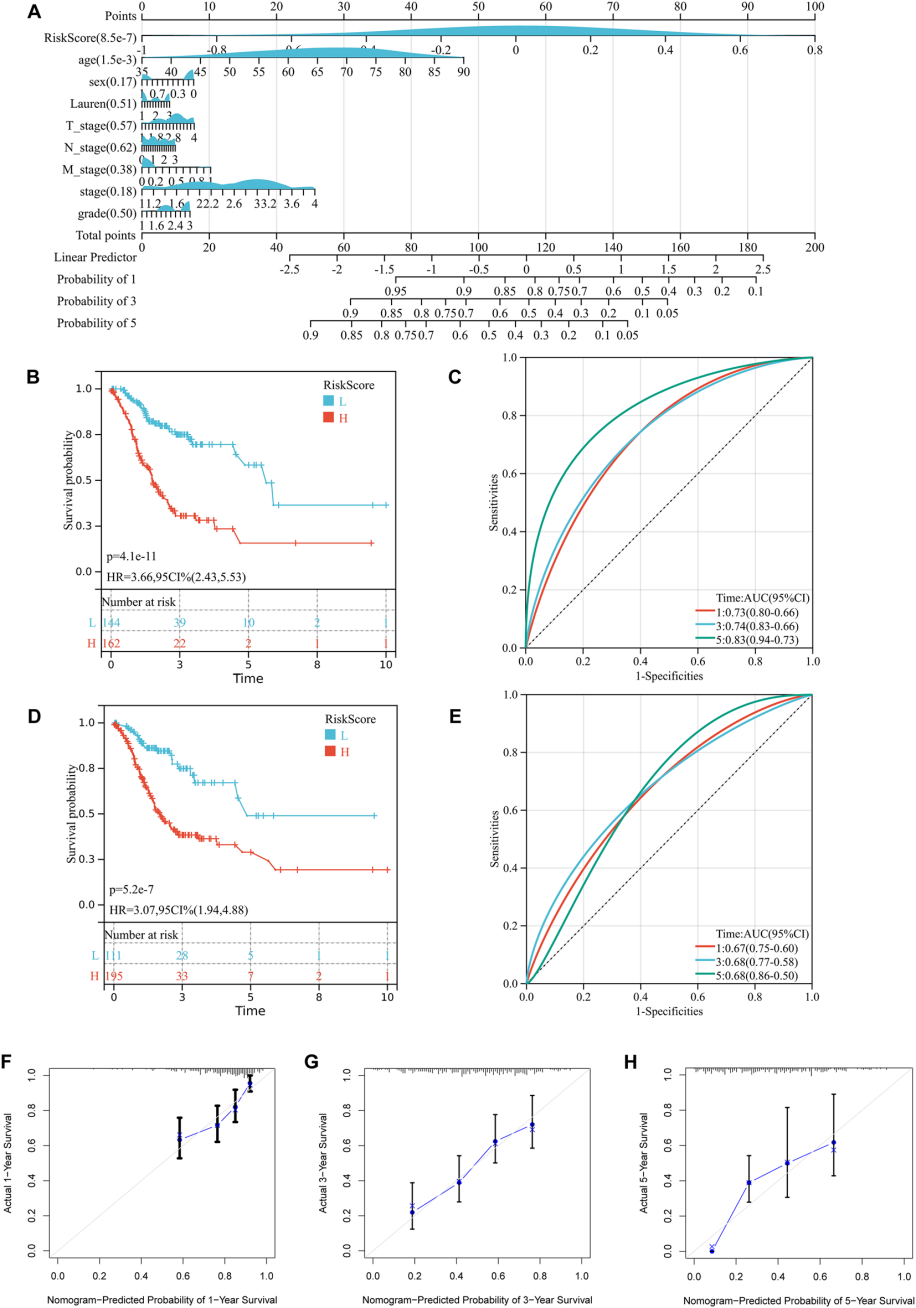
The nomogram was generated to improve risk stratification and estimate survival probability. (**A**) The comprehensive nomogram for predicting probabilities of gastric cancer patients with 1-, 3- and 5-year overall survival in TCGA-STAD dataset. (**B**) Kaplan–Meier analyses of overall survival for this nomogram. (**C**) Received operating characteristic analyses of 1-, 3- and 5-year overall survival for this nomogram. (**D**) Kaplan–Meier analyses of overall survival for the nomogram without hypoxia-related risk score. (**E**) Received operating characteristic analyses of 1-, 3- and 5-year overall survival for the nomogram without hypoxia-related risk score. (**F–H**) The calibration plots for predicting gastric cancer patients with 1-, 3- and 5-year overall survival in TCGA-STAD.

The HRRS, together with other clinical features, including age, Lauren, pathological T stage, pathological N stage, pathological M stage, stage and grade were enrolled as covariates to perform the analysis. We constructed a nomogram that serves as a clinically relevant quantitative method by which clinicians can predict mortality in GC patients (Fig. 3A). In addition, we confirmed the prognostic value of the nomogram, which was found to be significantly associated with OS (Fig. 3B,C). At the same time, we also analyzed the prognostic value of the model after removing HRRS (Fig. 3D,E). In the calibration analysis, the prediction lines of the nomogram for 1-, 3- and 5-year survival probability were extremely close to the ideal performance (45-degree line) (Fig. 3F–H).

### Mutation, DNA methylation, gene enrichment pathway and immune cell infiltration characteristics of gastric cancers in different hypoxia-related risk group

**Figure 4:**
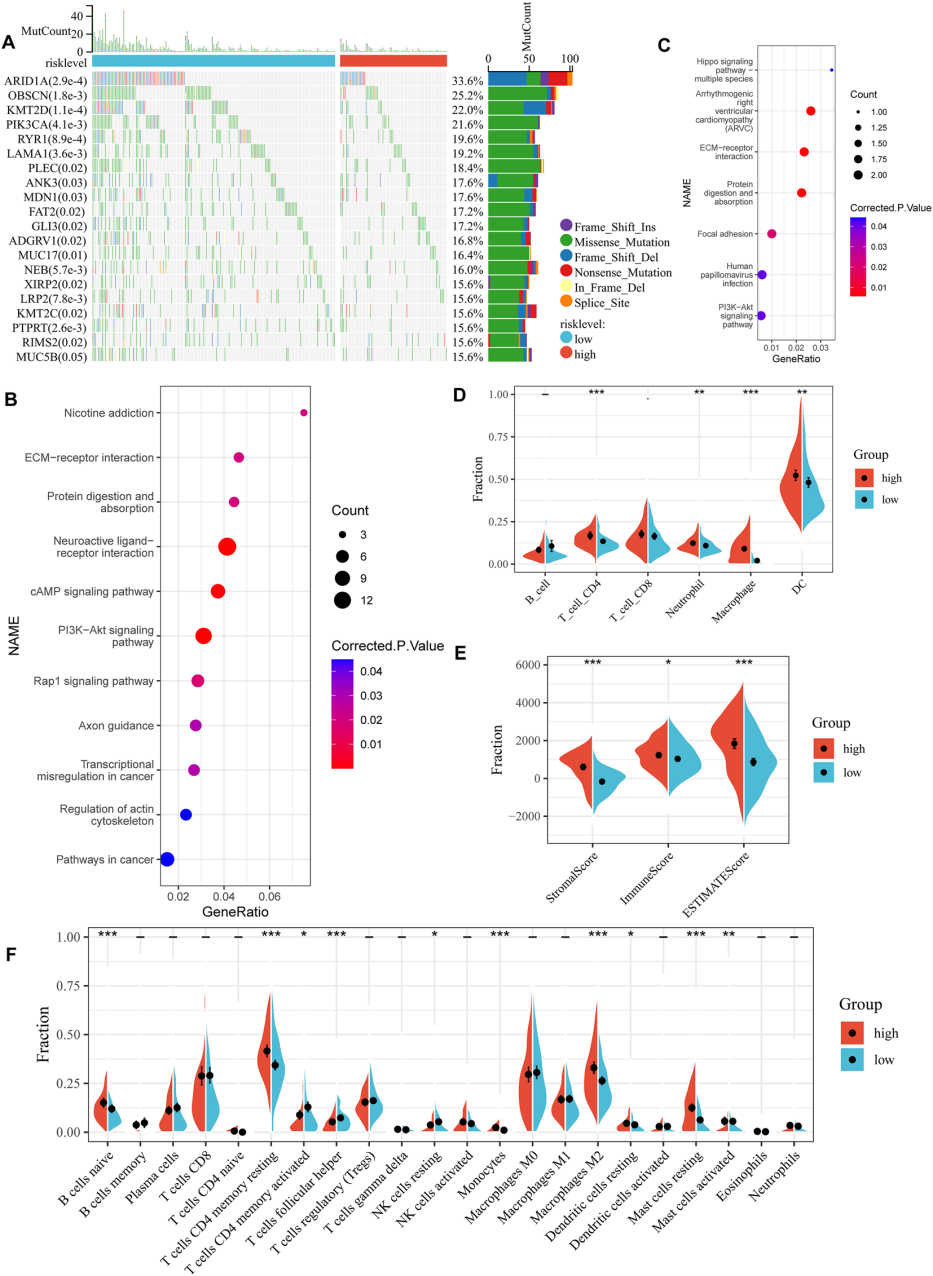
Differences between high- and low-risk groups. (**A**) Top 20 differentially mutated genes between two risk subgroups in all gastric cancer patients of TCGA-STAD cohort. (**B**) KEGG signaling pathway enriched for genes with low methylation and high expression in high-risk group. (**C**) KEGG signaling pathway enriched for shared genes between low methylation and high expression in high-risk group and turquoise module genes. (**D**) Relative proportion of 6 infiltrating immune cells estimated by TIMER between two risk subgroups of TCGA-STAD cohort. (**E**) Stromal score, Immune score and ESTIMATE score between two risk subgroups of TCGA-STAD cohort. (**F**) Relative proportion of 22 infiltrating immune cells estimated by CIBERSORT between two risk subgroups of TCGA-STAD cohort.

As shown in Fig. 4A, among the top 20 mutated genes, the low-risk group appears to have a higher mutation rate relative to the high-risk group. This may be the reason why the low-risk group has a larger sample size. Three of the top 20 mutated genes in gastric cancer differed between the two groups (Fig. 4A and Fig. S4).

For DNA methylation analysis, in order to make the analysis results more credible, we removed CpG sites that were both hypermethylated and hypomethylated in both high-risk and low-risk groups. Then, the difference beta value is set to 0.15. Because DNA hypermethylation inhibits DNA transcription and corresponding hypomethylation promotes DNA transcription, we performed differential expression analysis between the two subgroups using DESeq2. Finally, 183 low-methylation and high-expression genes were obtained in the high-risk group, while only 2 low-methylation and high-expression genes were obtained in the low-risk group (Table S5). Hypomethylated and highly expressed genes in the high-risk group were significantly associated with multiple tumor-related pathways, such as PI3K-Akt signaling pathway, cAMP signaling pathway, Rap1 signaling pathway, ECM-receptor interaction and so on (Fig. 4B). Interestingly, we found that these genes share 17 genes with the hypoxia-related turquoise module genes described earlier. And the Kyoto Encyclopedia of Genes and Genomes (KEGG) pathways enriched by these 17 genes are also mostly tumor-related pathways (Fig. 4C).

We then used the GSEA method to analyze KEGG pathway of different risk groups. GSEA results showed that as many as 105 KEGG pathways were enriched in the high-risk group, while only 11 pathways in the low-risk group (Tables S6, S7). The high-risk group was enriched for tumor related pathways such as focal adhesion, TGF-beta signaling pathway, PI3K-Akt signaling pathway, cell adhesion molecules, gastric cancer, JAK-STAT signaling pathway, microRNAs in cancer, Hippo signaling pathway, Wnt signaling pathway, and others, but the low-risk group did not have enrichment for tumor related pathways. This also proves that the high-risk group has rich tumor characteristics. In addition, we separately calculated 10 tumor-related pathways and 50 tumor hallmark pathways scores in the TCGA-STAD cohort, and found that the vast majority of pathways were more highly expressed in the high-risk group (Fig. S5).

Accumulating evidence suggests that hypoxia is an important feature of tumors that can modulate the tumor’s immune response. TIMER database showed that CD4 T cells, Neutrophil, macrophages and DC cells are highly infiltrated in high-risk groups (Fig. 4D). Immuno-infiltration analysis showed that high-risk group had the higher immune-microenvironment infiltration in the TCGA-STAD, followed by low-risk group had the lower immune-infiltration score (Fig. 4E). CIBERSORT along with the LM22 matrix was used to assess immune cell infiltration in the low- and high-risk groups of TCGA-STAD. Nine types of cells were different between the two groups, and only T cells CD4 memory activated, T cells follicular helper and NK cells resting were highly infiltrated in the low-risk group (Fig. 4F). We then found that multiple immune cells associated with tumor progression, including: M2 macrophages, dendritic cells, and mast cells, had higher level infiltration in the high-risk group. Perhaps these immune cells enabled the high-risk group of gastric cancer cells to achieve their purpose of immune escape (Fig. 4F). The high infiltration of T cells CD4 memory resting in the high-risk group may be the result of a compensatory increase after the suppression of immunity by these myeloid cells (Fig. 4F). But this needs to be verified by subsequent experiments.

### HRRS–based treatment strategy for gastric cancer

**Figure 5:**
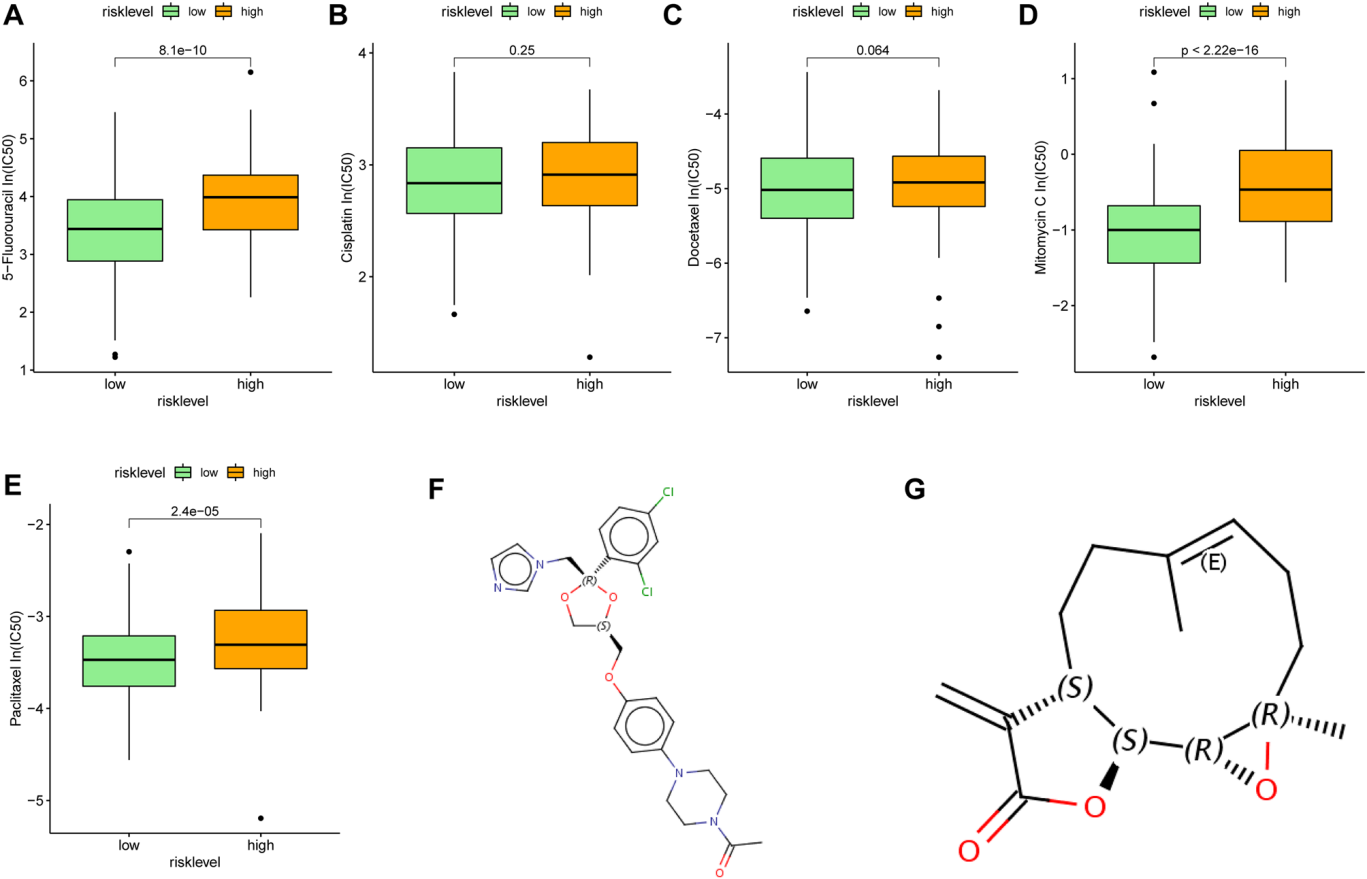
The estimation of chemotherapy response and potential therapeutic drugs for gastric cancer. (**A–E**) The chemotherapy response of two metabolic subtypes for 5 common chemotherapy drugs. (**F,G**) The molecular structure of the 2 small-molecule drugs for gastric cancer (F, ketoconazole; G, parthenolide).

The Cancer Genome Project (CGP) database was used to predict chemotherapeutic response. In CGP, we found 5 commonly used chemotherapy drugs for gastric cancer, but only three of them had significant differences in the estimated IC50 between the two subgroups (Fig. 5A–E). The low-risk patients were more sensitive to the anticancer drugs 5 − Fluorouracil, Mitomycin C and Paclitaxel.

Small molecule drugs with therapeutic effects on GC were screened using CMap database. Based on up-regulated genes and down-regulated genes, we screened out 2 potential gene-targeting small molecule drugs (Fig. 5F,G).

## Discussion

Hypoxia is a common feature of tumors^8^. Hypoxia has broad effects on various biological processes, such as angiogenesis and metastasis^9–11^. Then, intact tumor tissue includes not only cancer cells, but also surrounding blood vessels, lymphatic vessels, fibroblasts, inflammatory cells, and extracellular matrix. Traditional sequencing analysis is a holistic analysis of the entire tumor tissue. In our study we parsed the hypoxia feature of tumor cells at single-cell resolution and found out the genes and pathways related to hypoxia-related tumorigenesis and development. As GC prognostic outcomes vary widely, it is important to develop a robust classifier based on hypoxia signatures to classify patients with different risks and outcomes, which is critical to maximize the benefits of personalized treatment and timely follow-up of.

Through single-cell analysis, we found that not only epithelial cells showed hypoxic characteristics, but myeloid cells and stromal cells showed a higher hypoxic state, which forced us to consider that hypoxia not only affects malignant epithelial cells, but more myeloid and stromal cells. Then myeloid and stromal cells further promote tumor progression. Many previous studies have shown that macrophages and fibroblasts are associated with many features of GC, such as tumor malignant progression, epithelial-mesenchymal transition, and chemotherapeutic drug resistance^12–16^. But how hypoxia affects myeloid cells and stromal cells, and how they contribute to tumor progression, remains unclear. This is a good research direction and needs to be confirmed in our future research. Then we extracted the gene expression matrix of epithelial cells and performed WGCNA. WGCNA results are more reliable due to the large number of epithelial cells. Both hypoxia-related gene sets were significantly associated with the turquoise module, which also proved the reliability of the analysis results. The turquoise module genes were also enriched in many tumor-related pathways, suggesting that these pathways may be associated with hypoxia. By lasso cox method, we got a signature consisting of 13 genes for predicting prognosis. The 13-gene prognostic model has good predictive performance both in the training dataset and in multiple independent validation datasets. After removing the influence of other clinical characteristics, we found that the risk score derived from this model was an independent prognostic factor, and it was able to significantly increase the predictive power.

Both GSEA and single sample gene set enrichment analysis (ssGSEA) methods demonstrated that the high hypoxia score group was associated with more enrichment of tumor-related pathways, as expected. By analyzing the tumor-infiltrating immune cells predicted by the CIBERSORT algorithm, we found that among the 9 differentially infiltrating immune cells, only T cells CD4 memory activated, T cells follicular helper and NK cells resting were highly infiltrated in the low-risk group. We speculated earlier that macrophage hypoxia may play a role in tumor progression, which is consistent with the high infiltration of M2 macrophages in the high-risk group.

In the genomic mutation analysis, we found that the low-risk group instead had more mutations, which may be due to the larger sample size of the low-risk group. Epigenetic analysis indicated that high risk had more genes with both hypomethylation and high expression, and these genes were enriched in multiple tumor-related pathways. So, we thought that tumor hypoxia might affect the DNA methylation of cells and promote the transcription of genes associated with malignant features, thereby promoting tumor progression.

We predicted the therapeutic effects of 5 common chemotherapeutic agents in different hypoxia risk score subtypes. Patients in the low-risk group were more sensitive to three of the five chemotherapy drugs. We also predicted possible 2 potential gastric cancer drugs based on differentially expressed genes in high and low risk groups. This allows medical staffs to more accurately select a more suitable therapy program for patients.

## Conclusion

Our study illustrates the crucial role of hypoxia in GC. Hypoxia-related gene prognostic model has been established and has good performance. HRRS can also be used to guide a patient's drug treatment strategy.

## Materials and methods

### Data acquisition and processing

We systematically searched publicly available gene expression datasets from GC. After removing datasets with no prognostic survival information, a total of 6 datasets come from the Gene Expression Omnibus (GEO; https://www.ncbi.nlm.nih.gov/gds/) (GEO: GSE62254^17^, GSE15459^18^, GSE57303^19^, GSE34942^20^, GSE84437^21^ and GSE26942^22^, and an RNA-sequencing dataset (TCGA-STAD) from The Cancer Genome Atlas (TCGA; https://portal.gdc.cancer.gov/) were found. Four datasets (GSE62254, GSE15459, GSE57303, and GSE34942) from the GPL570 platform were combined into one dataset, named the GPL570 metadata set, using the “oligo” package in R^23^. The TCGA-STAD count expression data files and clinical data were downloaded using the “TCGAbiolinks” software package in R^24^. RNA-sequencing count values were converted to transcripts per million (TPM) values. The TCGA-STAD somatic mutation and the DNA methylation profile of the illumina human methylation 450 platform were downloaded using the R package “TCGAbiolinks”, and the somatic mutation data were analyzed using the R package “maftools”^25^. Methylation analysis was performed using the R package "ChAMP" ^26^. It is generally considered that a β value greater than 0.6 is fully methylated, 0.2–0.6 is partially methylated, and less than 0.2 is completely unmethylated. For differentially methylated probes (DMPs) analysis, we first removed fully methylated and fully unmethylated CpG sites in high-risk group and low-risk group and |diffBeta| is set to 0.15.

### Screening of hypoxia-related genes

To explore the characteristics of hypoxia at the single-cell level, we downloaded the GSE183904 single-cell dataset from the GEO database. Genes expressed in more than three cells and cells expressed in more than 300 genes were considered for subsequent analysis. Cells with mitochondrial RNA percentages of > 20 were filtered out. We use the “DoubletFinder” package to remove the “doublets cell”^27^. We used principal component analysis (PCA) to perform dimensionality reduction and then perform cluster analysis, and perform cell annotation based on marker genes of different types of cells.

According to the epithelial cells annotated by gene markers, we extracted the normalized expression matrix of the top 5000 highly variable genes. To find modules of highly correlated with hypoxia, WGCNA was performed using the WGCNA R package^28^ and carried out on top 5000 highly variable genes. Finally, modules that were significantly associated with hypoxia traits were selected for further analysis.

### Construction of hypoxia related risk model

Based on the hypoxic-related module genes obtained by single-cell analysis, we obtained the expression files of the corresponding genes of the TCGA-STAD dataset transformed by log2 and the z-score. Then, we used the R software package “glmnet” to perform the lasso-cox analysis. In addition, we also set up tenfold cross-validation to obtain the optimal model. Finally, a HRRS was constructed: HRRS = ∑(C × EXP), where EXP is the expression value of the gene and C is the regression coefficient for the corresponding gene in lasso Cox model.

### Hypoxia signature model validation

The samples of the TCGA-STAD dataset were divided into high-risk and low-risk groups based on the calculated hypoxia scores. The optimal cut-off value was determined through the R package “maxstat”. The minimum sample number is set to greater than 25%, and the maximum sample number is set to less than 75%. The KM method with log-rank test was used to further analyze the prognostic differences between the two groups. To evaluate the predictive efficiency of the hypoxia risk signature in the 1-, 3-, and 5-years survival rate, we performed the received operating characteristic (ROC) curve and AUC. We integrated prognostic and clinicopathological features to construct a nomogram to visually assess the patient's 1-, 3- and 5-year survival rate in TCGA-STAD.

### Gene enrichment analysis and gene set enrichment analysis

KEGG pathway enrichment analyses were performed using KOBAS 3.0 online database^29^. We download the latest KEGG pathway data using R package “KEGGREST” and performed enrichment analysis sing the R package “clusterProfiler” to obtain the results of gene set enrichment^30^. We downloaded hallmark gene sets from MSigDB^31^. We downloaded the GSEA software (version 4.3) from the gene set enrichment analysis (GSEA: http://software.broadinstitute.org/gsea/index.jsp) website. NOM p-value < 0.05 were considered statistically significant. To evaluate the gene set enrichment level of individual samples, ssGSEA was adopted through the GSVA package^32^.

### Evaluation of infiltrating immune cells in the TME

The proportions of 22 immune cell types in GC samples were estimated using the CIBERSORT algorithm (https://cibersortx.stanford.edu/) with batch-corrected mode, relative mode and 1000 permutations of b mode^33^. Stromal cells and immune cells in tumor tissue were estimated using the ESTIMATE algorithm^34^. TIMER is also used to assess the proportions of six types of immune cells^35^. Wilcoxon test was used to difference test.

### Additional bioinformatic and statistical analyses

The DESeq2 package in R was used to identify the differentially expressed^36^. Differences between the two groups were compared using Wilcoxon test. The ANOVA is used to detect the differences between multiple groups. The half maximal inhibitory concentration (IC50) is estimated by R package “pRRophetic”^37^. The Connectivity Map (CMap, https://clue.io/) was used to predict the small candidate molecules based on differentially expressed genes. All of the above analyses were performed using the R software (version 4.0.2, http://www.rproject.org). Statistical differences not specifically stated were set at p < 0.05.

## Supplementary Information

Supplementary Figure S1.

Supplementary Figure S2.

Supplementary Figure S3.

Supplementary Figure S4.

Supplementary Figure S5.

Supplementary Tables.

## Data Availability

The data that support the findings of this study are available in GEO (https://www.ncbi.nlm.nih.gov/geo/, GSE62254, GSE15459, GSE57303, GSE34942, GSE84437, GSE26942 and GSE183904), TCGA (https://portal.gdc.cancer.gov/repository, TCGA-STAD), and the Supporting Information.

## Abbreviations

GC: Gastric cancer
GEO: Gene expression omnibus
TCGA: The cancer genome atlas
TPM: Transcripts per million
DMPs: Differentially methylated probes
PCA: Principal component analysis
WGCNA: Weighted gene co-expression network analysis
HRRS: Hypoxia-related risk score
KM: Kaplan–Meier
ROC: Received operating characteristic
AUC: The area under the curve
KEGG: The Kyoto encyclopedia of genes and genomes
GSEA: The gene set enrichment analysis
ssGSEA: Single sample gene set enrichment analysis
IC50: The half maximal inhibitory concentration
