# Supplementary figures and images for: Comprehensive analysis of hypoxia-related genes for prognosis, immune features, and drugs treatment strategy in gastric cancer using bulk and single-cell RNA-sequencing
Source: Sci Rep. 2022 Dec 16;12:21739. doi: 10.1038/s41598-022-26395-5 (PMC9758178; doi:10.1038/s41598-022-26395-5)

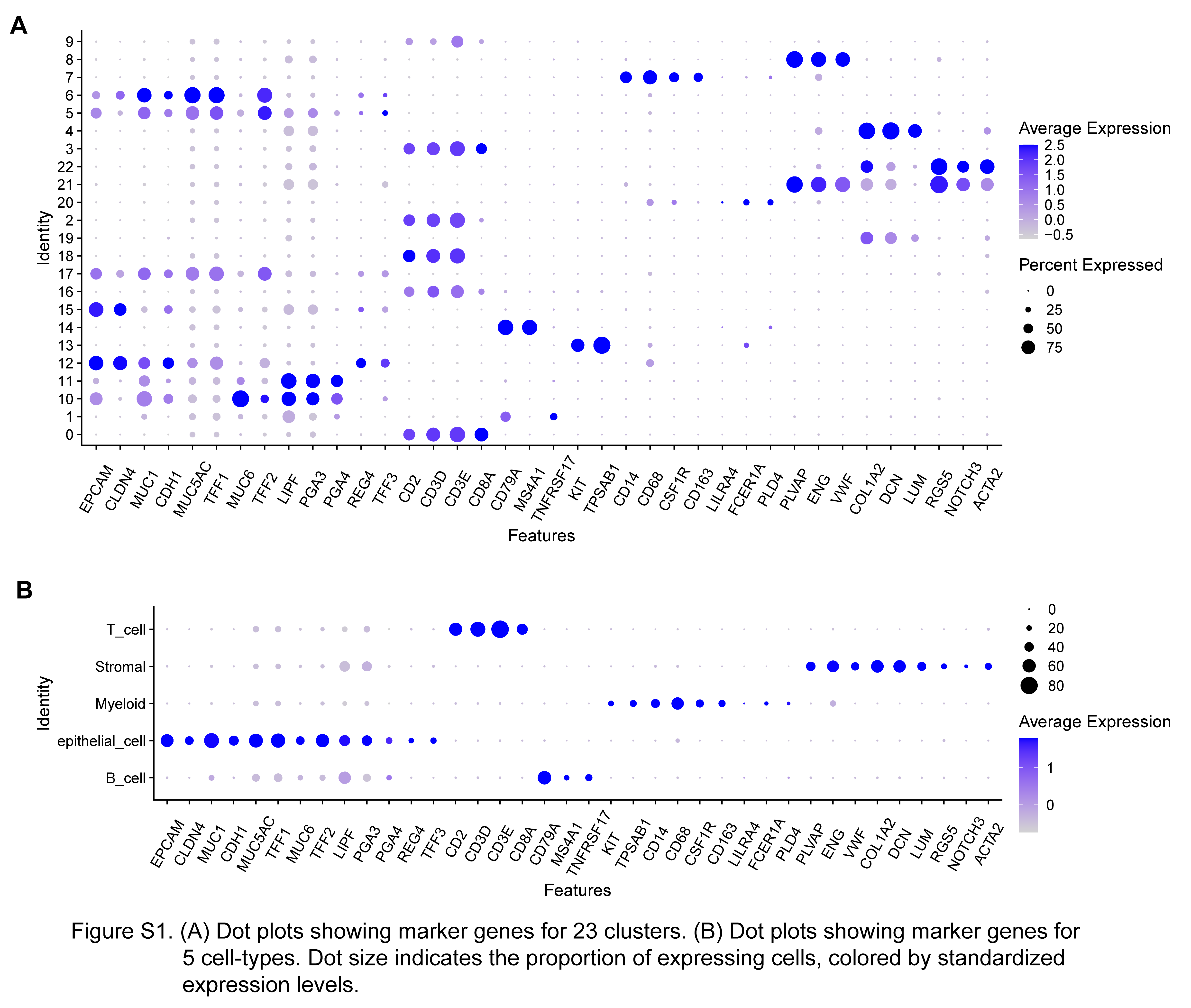

Supplement: Supplementary file 1 — Supplementary Figure S1. [file 41598_2022_26395_MOESM1_ESM.tif]

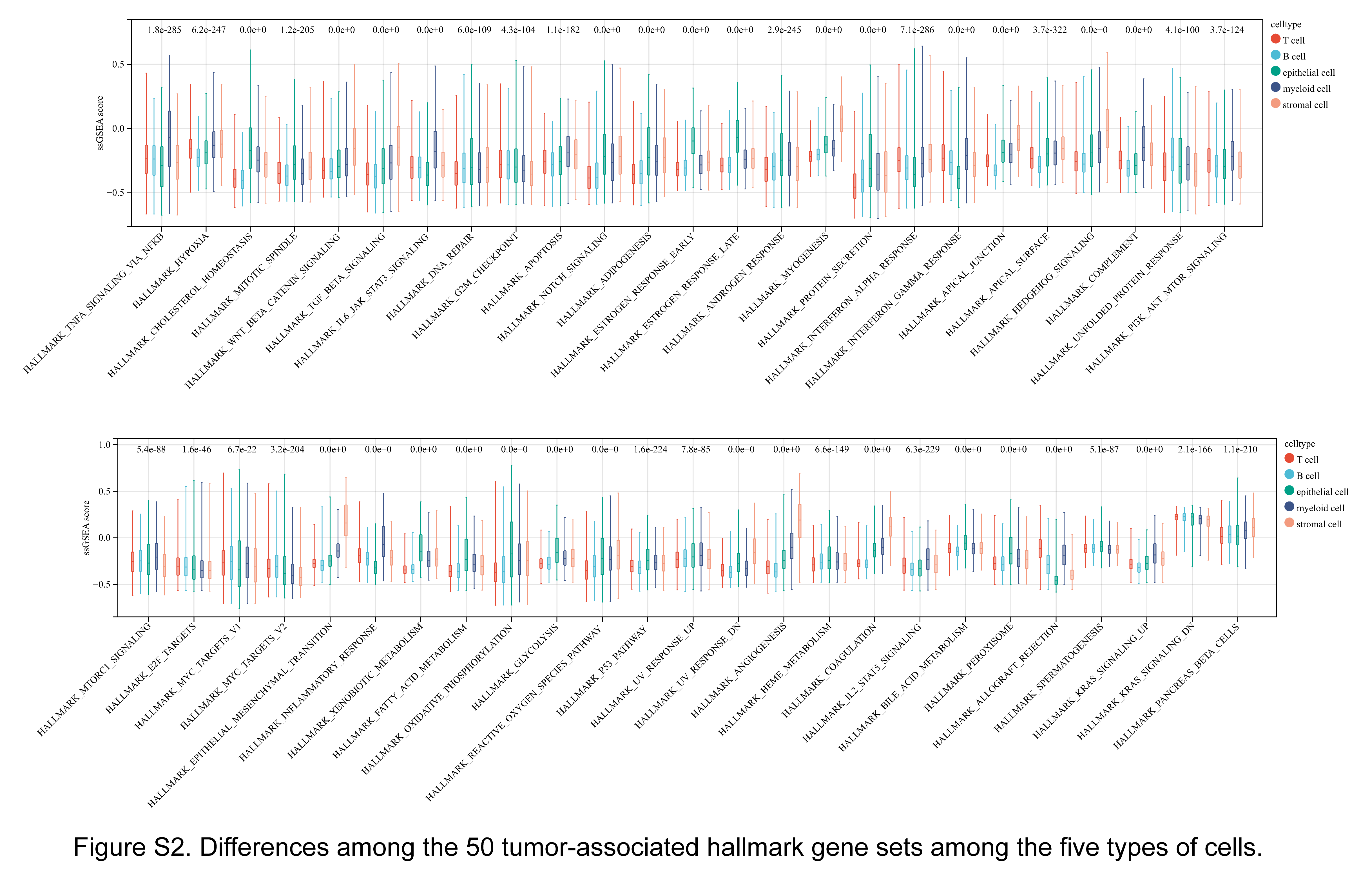

Supplement: Supplementary file 2 — Supplementary Figure S2. [file 41598_2022_26395_MOESM2_ESM.tif]

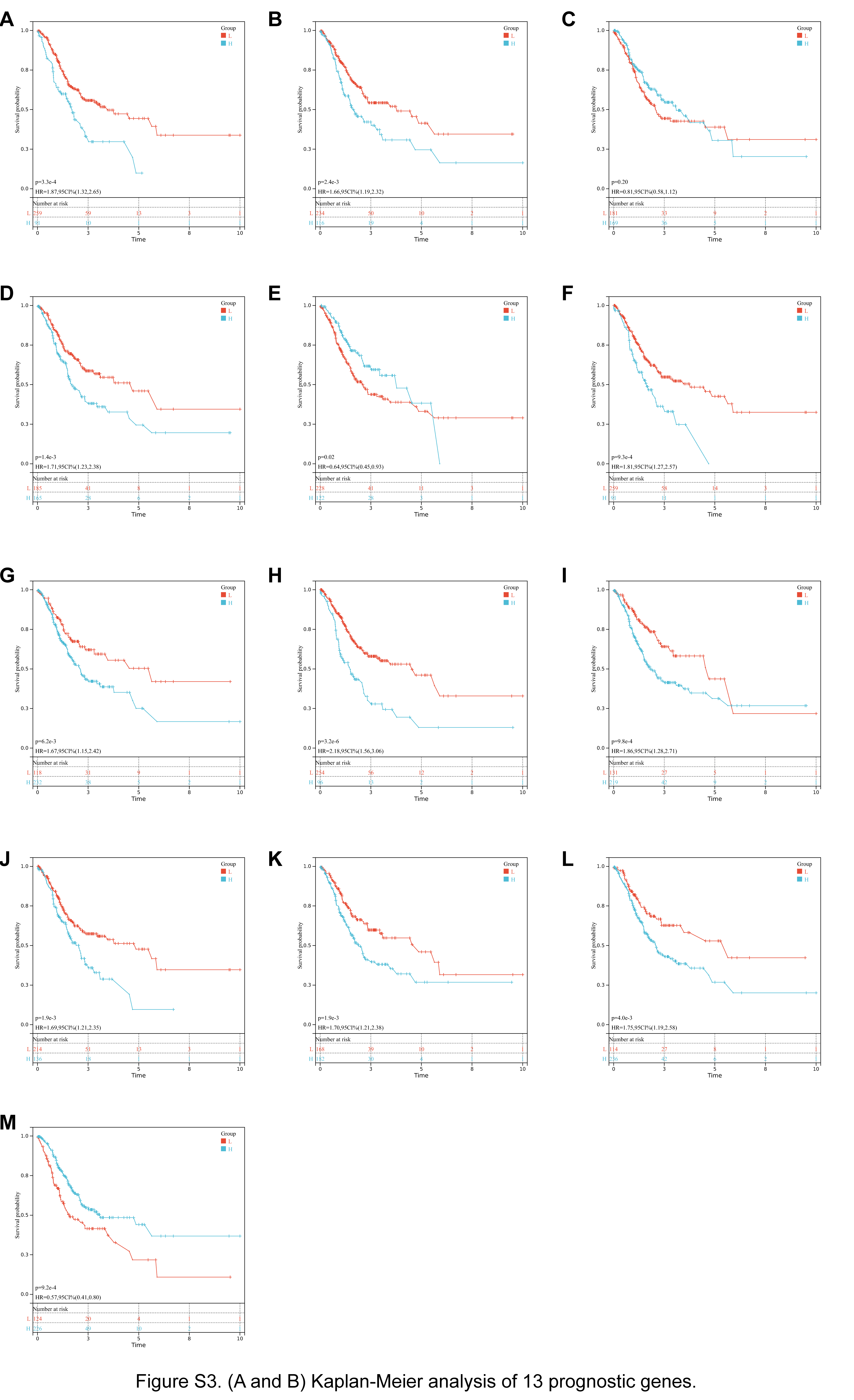

Supplement: Supplementary file 3 — Supplementary Figure S3. [file 41598_2022_26395_MOESM3_ESM.tif]

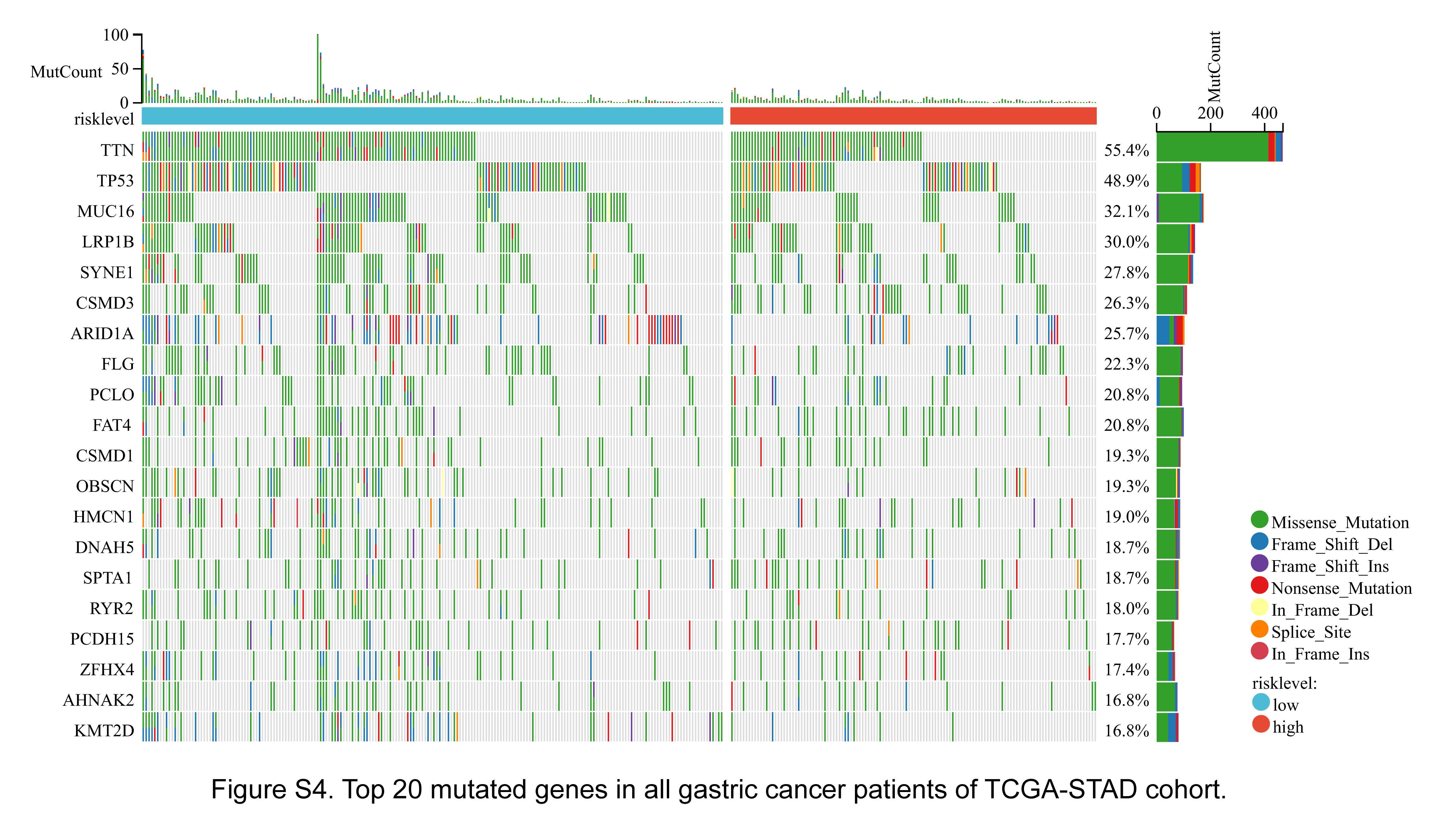

Supplement: Supplementary file 4 — Supplementary Figure S4. [file 41598_2022_26395_MOESM4_ESM.tif]

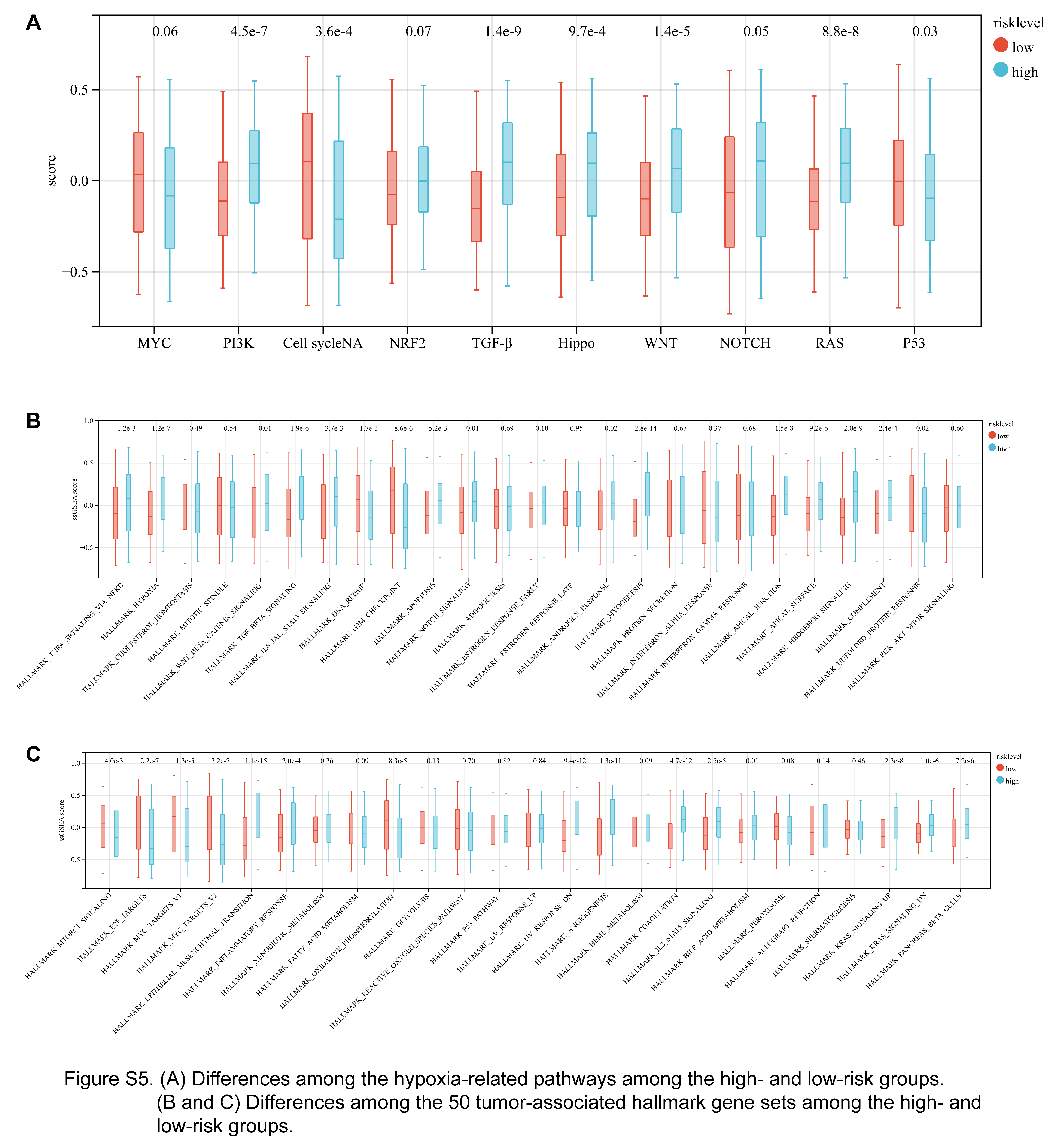

Supplement: Supplementary file 5 — Supplementary Figure S5. [file 41598_2022_26395_MOESM5_ESM.tif]
